# The development of proliferative verrucous leukoplakia in oral lichen planus. A preliminary study

**DOI:** 10.4317/medoral.20832

**Published:** 2016-03-31

**Authors:** María-José Garcia-Pola, Santiago Llorente-Pendás, Manuel González-Garcia, José-Manuel García-Martín

**Affiliations:** 1 Associate Professor of Oral Medicine. Faculty of Medicine. Catedrático José Serrano. 33006. Oviedo University. Spain; 2 Medical Doctor. Oral and Maxillofacial Surgeon. Private practice of Oral and Maxillofacial Surgery. Medical Director of Head and Neck Center. Pérez de la Sala 37.33007. Oviedo. Spain; 3 Assistant Professor of Oral and Maxillofacial Surgery. Faculty of Medicine. Catedrático José Serrano. 33006. Oviedo University. Spain; 4 Assistant Professor of Oral Health & Preventive Dentistry. Faculty of Medicine. Catedrático José Serrano. 33006. Oviedo University. Spain

## Abstract

**Background:**

Was to describe 14 cases of a proliferative verrucous leukoplakia as a clinical evolution of oral lichen planus.

**Material and Methods:**

The clinical and histopathological characteristics of 14 cases of OLP that progress towards a plaque-like and verrucous form were indicated, with monitoring over a period of six to 24.3 years.

**Results:**

The female/male ratio was 11/3, (78.6 and 21.4%). The mean age when the first biopsy was undertaken was 56.4 years old. None of the patients smoked during the study. As bilateral reticular was clinically diagnostic criterion, the second most frequent clinical form was the plaque form (n=10; 71.4%), followed by the atrophic (n=6; 42.8%), and erosive forms (n=4; 28.5%). Clinically it spread towards attached gingival mucosa and the hard palate. In the histopathologic study, there were a predominance of hyperkeratosis and verrucous epithelial hyperplasia. Three of the cases progressed to a squamous cell carcinoma, and one patient developed two verrucous carcinoma.

**Conclusions:**

Further research is needed to demonstrate if proliferative multifocal oral lichen planus and proliferative multifocal oral leukoplakia are the same disorder but have different behaviour of malignancy for reasons of origin.

**Key words:**Oral lichen planus, proliferative verrucous oral leukoplakia, malignant oral lichen planus, multifocal verrucous oral lichen planus, proliferative verrucous oral lichen planus.

## Introduction

Oral lichen planus and oral leukoplakia are the most common potentially malignant disorders. Oral lichen planus (OLP) is a chronic, autoimmune mucocutaneous disease, characterised by exacerbation and remission periods. OLP can present as white papules that gradually expand in a symmetrical manner to form a reticular pattern (Wickham’s striae), or as plaques, which may or may not come with atrophic, erosive, and bullous forms (1). Although oral leukoplakia (OL) has been defined as “predominantly white lesion of the oral mucosal that cannot be characterized as other definable lesion” (2), in some OLP patients, gingival location presenting as white papules or plaques may resemble leukoplakia (3). Even may at times be difficult to distinguish clinically from homogeneous and non-homogeneous leukoplakia (4).

The risk of development oral cancer in OLP patients has been the issue of much debate. The rate of transformation in individual studies ranged from 0 to 3.5% (5). The literature reveals that there may be a higher frequency of OLP transforming into squamous cell carcinoma in some locations, such as tongue, followed by buccal mucosa and gingival. With respect to the clinical form, results are somewhat arduous to interpret because carcinoma may be preceded by atrophic-erosive (6-8), non-erosive (9), in other series by plaque form (10), and finally there are published works that found equally atrophic-erosive or plaque form (11,12).

In 1985 Hansen *et al.* introduced a new subtype of OL designed as “proliferative verrucous leukoplakia” (PVL) (13). PVL is differentiated by slow-growing and progressive clinical course, with changes clinical and histopathologic. PVL is an aggressive form of oral leukoplakia with considerable morbidity and strong predilection to malignant transformation (14).

Guideline or protocols of diagnostic criteria have been published to provide diagnosis of PVL (15-17). In order to make the early and rapid diagnosis of PVL Cerero *et al.* (17) have suggested major and minor criteria that offer a great support, emphasizing the presence of histopathologic pattern such as hyperkeratosis verrucous hyperplasia or carcinoma.

Our aim was to describe the behaviour of oral lichen planus patients, characterised for presenting a clinical form that progressed to a proliferative verrucous plaque-like pattern, and monitored for a minimum period of six years by the same workgroup.

## Material and Methods

This descriptive observational study was conducted from a cohort of patients diagnosed of OLP, between September 1984 and February 2015. OLP patients were diagnosed by the same author clinically and by biopsy, based on the World Health Organization (WHO) criteria in 1978 (18) and on the criteria proposed by van der Meij and van der Waal in 2003 (1). The following requisites must be fulfilled: bilateral papules and reticular present, which may or may not come with other clinical forms of OLP (plaque, atrophic, erosive, bullous), and a histological description of band-like inflammatory infiltrate in the subbasal layer, liquefaction degeneration in the basal layer, and no dysplasia.

From OLP patients we included in the present study, those patients with the following progression behaviour: 1) predominating plaque-like form in almost a topographical area, with roughness and thickening, white-greyish in colour or verrucous form, 2) spreading multifocal growth affecting more than two oral locations and including the attached mucosa (gingival, alveolar process in edentulous area) or the hard palate, buccal or lingual mucosa, 3) histological criterion, through a second biopsy on the plaque-like or verrucous areas, with the presence of hyperkeratosis, acanthosis, papillomatous, or hyperplasia, without band-like inflammatory infiltrate (or without dominance), 4) treatment resistant, 5) monitoring for at least 5 years by the same doctor.

The studied variable were: gender, age, toxic habits (smoking, alcohol, and narcotics), location of the OLP, clinical form of OLP, affectation of other mucosa, appendages and skin, length of the monitoring, OLP treatment, microscopic changes in the second biopsy, and type of malignancy, number of carcinomas developed and their respective treatments.

The medical treatment options for OLP were: 1) Topical corticosteroids (TC) with 0.05% Clobetasol Propionate in Orabase and in aqueous solution; 2) daily oral administration of prednisone (0.5 mg/kg weight/day) followed by every other day; 3) acitretin 25-50 mg/day orally; 4) topical retinoids 0.1% 13-cis-retinoic acid in orabase. The excision treatment involved an intervention using a scalpel and CO2 laser surgery. The laser wavelength was 10.600nm and the vaporisation occurred between 4.5-7W. Treatments for the carcinomas included scalpel surgery, radiotherapy and laser CO2. Nystatin and fluconazole were used as an adjuvant treatment for complicated cases with secondary oral candidosis.

This project was approved by the Ethics in Research Committee from the University Central Hospital of Asturias.

## Results

Between years mentioned we recruitment a total of 515 oral lichen planus, from those, we collected a total of 14 patients (2.7%) that development proliferative verrucous leukoplakia.

The details of the demographic study for each of the 14 patients are summarised in  Table 1 and  Table 1Continue. The patients were monitored for a minimum of 6.3 years and a maximum of 24.3 years, with a mean follow up time of 14.5 years.

**Table 1:**
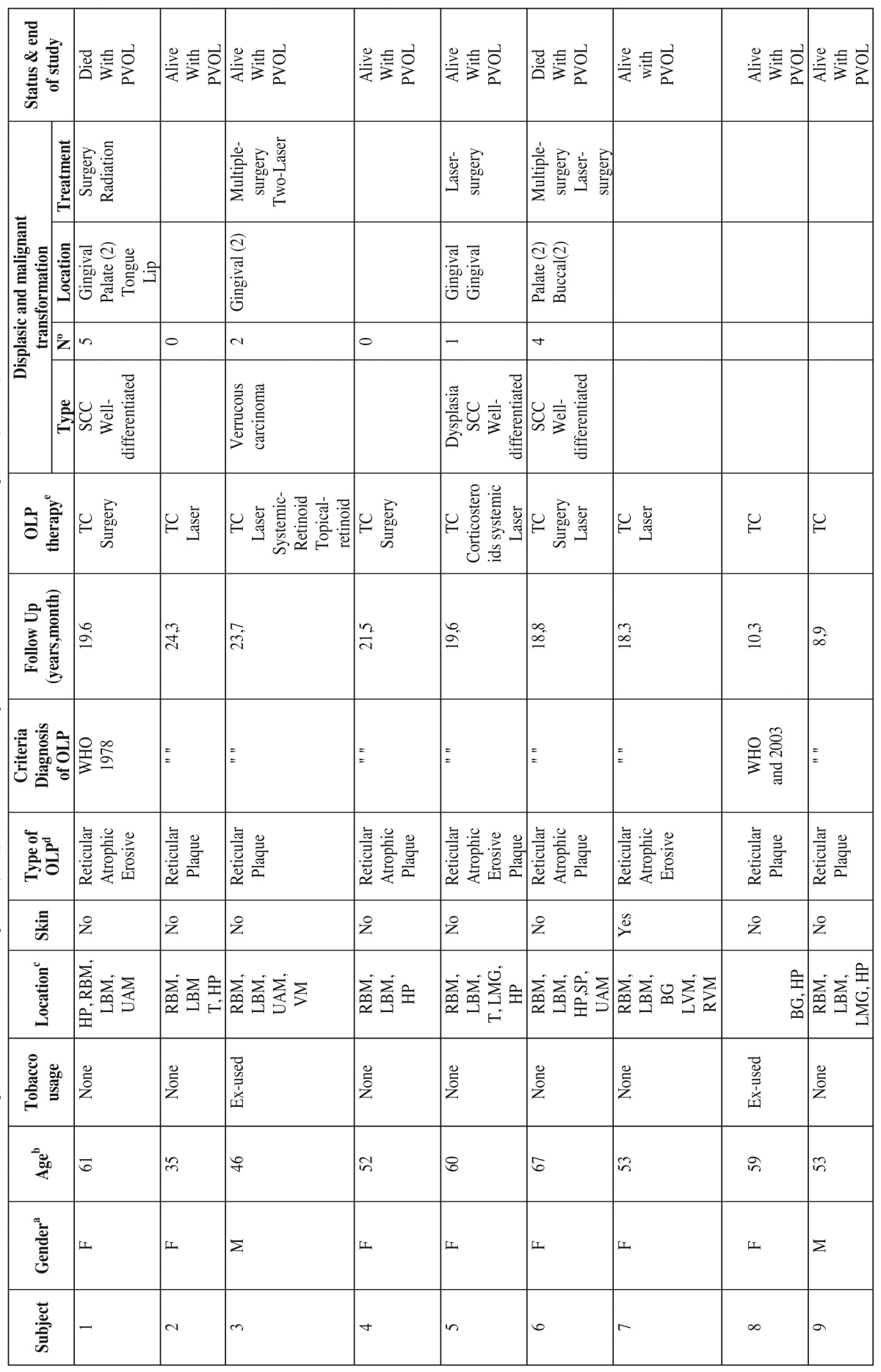
Clinical characteristics of the patients with oral lichen planus (OLP) that evolved into a proliferative verrucous oral leukoplakia (PVOLP).

**Table 1Continue:**
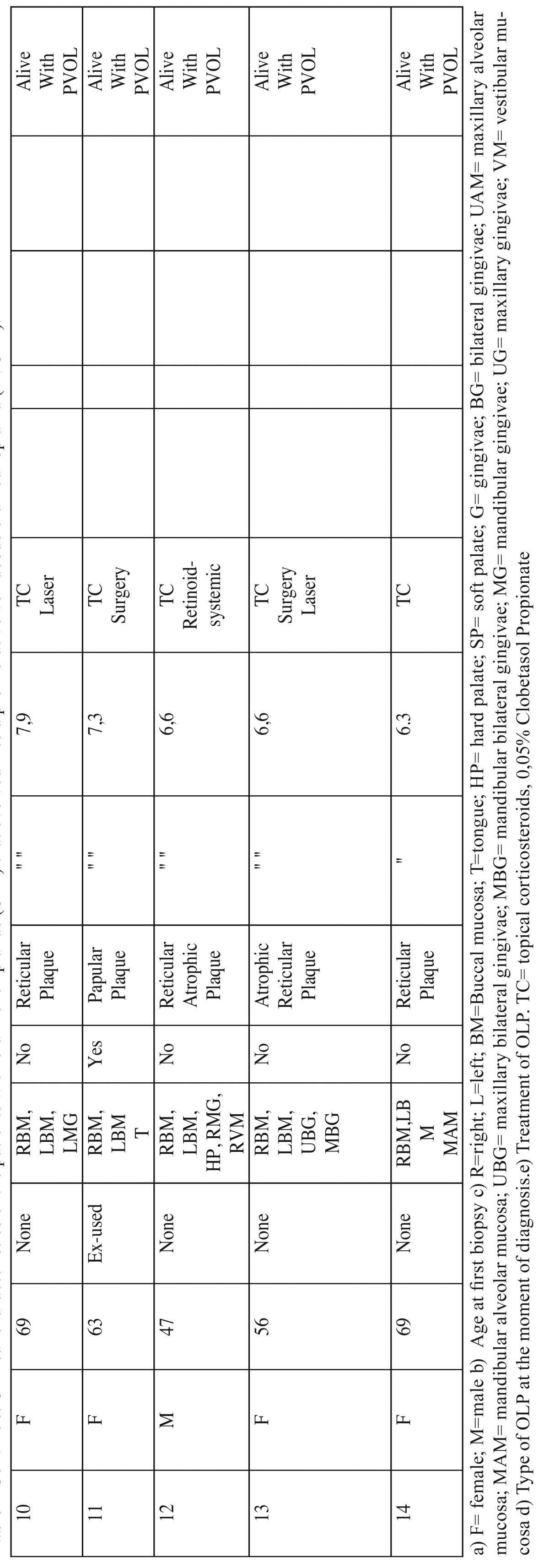
Clinical characteristics of the patients with oral lichen planus (OLP) that evolved into a proliferative verrucous oral leukoplakia (PVOLP).

The female/male ratio was 11/3, (78.6 and 21.4%). The mean age when the first biopsy was undertaken was 56.4 years old, 58.5 years old and 48.6 years old for women and men respectively.

None of the patients smoked or took narcotics during the study. Three were ex-cigarette smokers of more than 15 years. Three male patients drank one or two glasses of wine at the weekend. The women did not drink.

The predominant initial clinical together bilateral reticular form was the plaque form (n=10; 71.4%), followed by the atrophic (n=6; 42.8%), and erosive forms (n=4; 28.5%).

**Figure 1:**
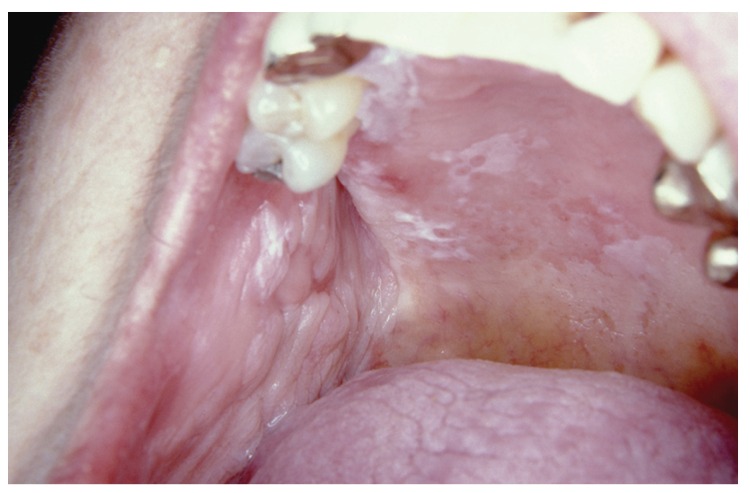
Reticular, plaque and atrophic type of lichen planus in hard palate, and reticular-erosive in buccal mucosa at the moment of diagnosis.

The most common location at baseline was the bilateral buccal mucosa (n=11; 78.5%), followed by the gingiva and palate mucosa (n=11; 78.5%), and the lingual mucosa (n=3; 21.4). They all had three or more affected areas (Fig. 1). Only two female patients had affected skin, which responded positively to treatment with topical corticosteroids.

The initial OLP treatment was topical corticosteroids in all cases. The remaining treatment options for each patient are detailed in  Table 1 and  Table 1 Continue. The patients did not show adverse haematological reactions.

**Figure 2:**
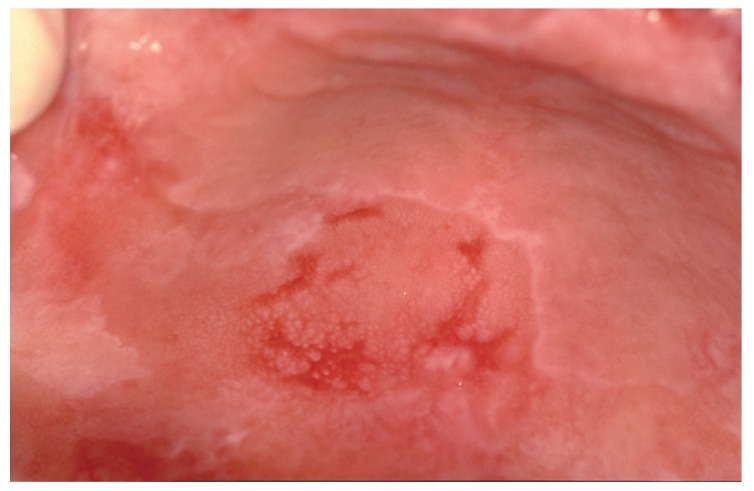
The same woman three years later with papular-reticular and erosive lichen planus in hard palate.

**Figure 3:**
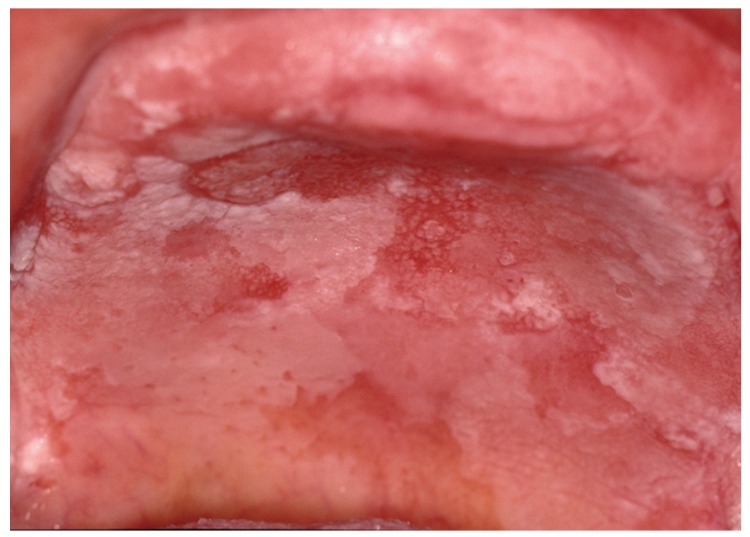
Verrucous lichen planus in hard palate and left upper buccal sulcus in the same patient after eigth years from initial diagnosis.

During the follow-up of patients, nine of them acquired clinical forms different from the initial (Fig. 2). Since the time of diagnosis, the mean time for developing plaque-like and multifocal verrucous lesions as the predominant lesion was 4 years (Fig. 3).

**Figure 4:**
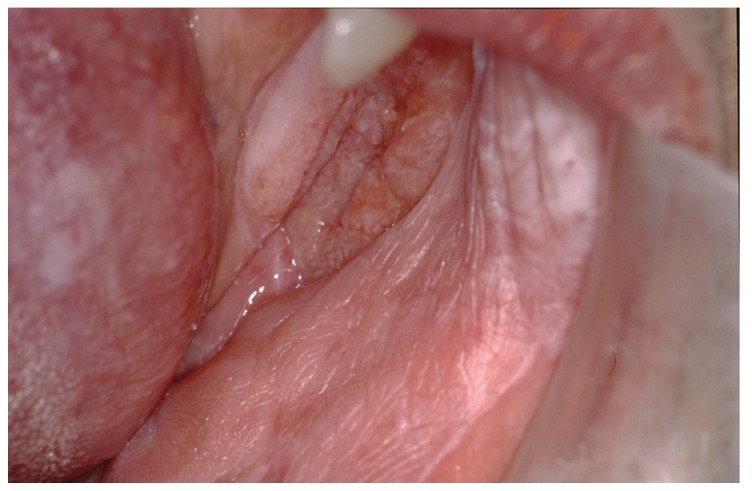
The same patients, ten years after initial diagnosis of OLP and two years after fast-growing multifocal and verrucous, showing the clinical appearance of squamous cell carcinoma in the gingiva and left lower buccal sulcus. She also presented plaque OLP in gingiva, buccal and dorsum of the tongue.

Hyperkeratosis (parakeratin and or orthokeratin) was present in part of all specimens, acanthosis and papillomatous squamous proliferation partly in ten cases, and verrucous hyperplasia in nine cases. Four patients (28.5%) went on to become malignant, three cases of squamous cell carcinoma (SCC) were well-differentiated, and one verrucous carcinoma. The mean time to malignant transformation from the first diagnosis was 9 years. Three cases were female and one male. The most common location of carcinoma was attachment mucosa (Fig. 4), gingival and hard palate.

## Discussion

In the present study we describe the characteristics of PVL in patients previously diagnosed of oral lichen planus, contributing to corroborate what in recent reports have suggested, the clinical relationship between OLP and PVL (19) and the presence of local histopathologic pattern with lymphocytic infiltrate in some areas of PVL (20)

The exact prevalence of PVL is not well known, most cases reported about long time series, its appearance, behaviour, treatment and malignant transformation. In our study we present, from a set of OLP diagnosed at first visit by the same professional, a total of 2.7% patients that suffered clinical and histopathologic changes towards PVL. Brzak *et al.* (21) contributed data of 0.15% OLP joints OL in a sample that was performed during ten years. To avoid future discrepancies, our figures did not include patients who had the first day OL lesion with OLP.

With regard to possible evolution and change of the clinical form of OLP, Carbone *et al.* (12) observed that 15% patients manifested changes from red lesions to white ones with and without treatment. Furthermore, in a recent study, Chainani *et al.* (22) have described nine cases of oral leukoplakia in patients diagnosed previously of OLP, in a follow-up between two and ten years. Therefore, whether OLP can change their clinical form towards white type, the plaque-like form, the most frequent in our sample (71.4%) at the moment of diagnoses, could be the starting point to take into account in the early diagnosis of PVL derivative from OLP.

In addition, it is difficult to map and quantify the subtype given that OLP is very active in terms of its clinical forms and extension (23), and because most of patients can have numerous locations (24). In this sense, we found the same prevalence in relation to location in buccal and gingival mucosa and all patients were affected in three or more areas, and one of those always was attached mucosa (gingival or palate). From this point of view, the location of attached mucosa may play an important role in early diagnosis of PVL.

Scientific literature evidences to confirm clinical OLP diagnosis is necessary to take a biopsy. Histological behaviour of the type of OLP that we described suffered significant changes from the initial diagnosis until it became a multifocal verrucous form. A limitation of this study was the misinformation from each biopsy performed for individual patients, we have referred to first biopsy to diagnose OLP and the biopsy which histhologically confirmed PVL with predominated in the epithelium hyperkeratosis, or/and verrucous hyperplasia, and SCC, and VC. All of the patterns shown in the study were based on the literature to diagnose of PVL (13,25).

Most authors emphasize that OLP treatment should focus on the symptom-based atrophic and erosive forms, considering that papule-reticular OLP does not require treatment (3). The first treatment option is a corticosteroid, although on reviewing the issue, it is stated that an effective treatment to cure OLP is lacking, and that no scientific evidence of a corticosteroid being more useful than any other in the topical treatment of OLP exists (26). The individualisation of the treatment was other of the limitations of our sample, given that on occasions, recurrence or relapse was so common along with treatment resistance that some of the patients needed immediate action to switch and combine different alternatives.

The use of topical retinoids was proposed for the reticular and plaque-like OLP forms, and as an alternative to treatment with corticosteroids (27). One-third of PVL patients improved after an average of 5-6 months with topic or systemic retinoid therapy (28). We proposed it orally and topically on two patients (cases 3 and 12), with topical corticosteroid and CO2 laser therapy applied during rest periods. CO2 laser vaporization has been proposed as a treatment alternative for erosive OLPs resistant to corticosteroid therapy (29), in plaque forms (30) and in PVL without dysplasia (14). CO2 laser was the treatment for seven of our patient cases (in combination with other treatments), and four of them showed recurrence, that resistant to treatment was a cause for concern. This is another considerable difference in treatment response between our proliferative verrucous form in OLP, and oral leukoplakia derived from OLP, described by Chainani-Wu *et al.* (22), because this oral leukoplakia treated with CO2 laser has been satisfactory evolution during their follow-up.

The potential for OLP to become malignant continues to be a very controversial issue, with its incidence fluctuating between 0 and 3.5 percent (5). The progression from reticular forms to leukoplakia-type observed in our patients established that the malignant behaviour was very similar to that described previously in the PVL when it comes to occurring predominantly in women and located on the gum (13) and alveolar crest (31). It is estimated that the 70% of PVL goes onto become malignant (25) and a 28.5% figure was found among our cases. This could give rise to the thought that OLP that progresses towards the multifocal verrucous forms become malignant in the same proportion as verrucous leukoplakias (non-proliferative) (32), and in a lower proportion than the proliferative verrucous leukoplakia (14).

Mignogna *et al.* (10) detected that almost all the forms of OLP that became malignant in their study were characterised by presenting keratinous, reticular and plaque-like areas. In line with this findings, we suggest that those clinical forms of OLP should be included in the progressive monitoring protocols and closely followed.

It is evident from other studies that OLP is seen more frequently in women and that long time series revealed that the use of tobacco and alcohol do not participate in the pathogenesis of OLP (12). For both reasons we do not recognise the sex, tobacco and alcohol as relevant factors in the early diagnosis of PVL developed from OLP.

The term “multifocal”, has now been reused by Aguirre-Urizar (33) for more stringent referencing of early PVL diagnoses, and given the similarity of behaviour of these lesions in ours cases, it turns out to be very appropriate term to use for the description.

Given the usefulness of having some criteria to diagnose of PVL (17), we propose some clinical major, and histological criteria, because the minor criteria indicated for PVL, predominantly female and non-smokers, may not be used as they are prevalent among the patients with OLP studied. On the basis of the above, we propose the following criteria to define this type of proliferative multifocal OLP: 1) predominance of the plaque-like form of OLP with thickening of the lesion at some point during the progression; 2) at least three affected topographical areas, given that two of them in symmetry would already be included in the OLP diagnosis, highlighting the location in attached mucosa (gums and palate) 3) resistance to medical and surgical treatment, 4) to remain during at least 5 years of monitoring those parameters; and 5) histological changes in the epithelial thickness (hyperkeratosis), verrucous hyperplasia, verrucous carcinoma or oral squamous cell carcinoma, a priori the most important criteria.

## Conclusion

Based on the different percentage of malignance in our sample (28.5%) and the present in the literature review, from 40% to 74% of PVL, additional long-term research is necessary to clear if proliferative multifocal lichen planus and proliferative multifocal oral leukoplakia are the same disorder but have different behaviour of malignancy for reasons of origin.
